# A deletion at the polled P_C_ locus alone is not sufficient to cause a polled phenotype in cattle

**DOI:** 10.1038/s41598-022-06118-6

**Published:** 2022-02-08

**Authors:** Sadie L. Hennig, Joseph R. Owen, Jason C. Lin, Bret R. McNabb, Alison L. Van Eenennaam, James D. Murray

**Affiliations:** 1 Department of Animal Science, University of CA – Davis, Davis, CA USA; 2 Department of Population Health and Reproduction, School of Veterinary Medicine, University of CA – Davis, Davis, CA USA

**Keywords:** Biotechnology, Genetics

## Abstract

Dehorning is a common practice in the dairy industry, but raises animal welfare concerns. A naturally occurring genetic mutation (P_C_ allele) comprised of a 212 bp duplicated DNA sequence replacing a 10-bp sequence at the polled locus is associated with the hornless phenotype (polled) in cattle. To test the hypothesis that the 10 bp deletion alone is sufficient to result in polled, a CRISPR-Cas9 dual guide RNA approach was optimized to delete a 133 bp region including the 10 bp sequence. Timing of ribonucleoprotein complex injections at various hours post insemination (hpi) (6, 8, and 18 hpi) as well as in vitro transcribed (IVT) vs synthetic gRNAs were compared. Embryos injected 6 hpi had a significantly higher deletion rate (53%) compared to those injected 8 (12%) and 18 hpi (7%), and synthetic gRNAs had a significantly higher deletion rate (84%) compared to IVT gRNAs (53%). Embryo transfers were performed, and bovine fetuses were harvested between 3 and 5 months of gestation. All fetuses had mutations at the target site, with two of the seven having biallelic deletions, and yet they displayed horn bud development indicating that the 10 bp deletion alone is not sufficient to result in the polled phenotype.

## Introduction

Dehorning and disbudding are common practices used in the beef and dairy industry to physically remove an animal’s horns. These procedures are done as preventative measures to protect both animals and handlers, however they are costly to the producer and painful to the animal. Recent studies have shown roughly half of producers that perform these procedures use some sort of pain management^1^. Taking the welfare of the animal into consideration, it has been proposed to eliminate the need for dehorning by introducing a naturally occurring hornless allele into elite horned cattle lines via genome editing to prevent horn bud development^2–5^.

A dominant allele (P) at the polled locus on *Bos taurus* chromosome 1 resulting in the hornless (polled) phenotype in cattle is common among some cattle breeds, such as Angus, but is rare in breeds such as Charolais and Holstein, especially in the elite breeding lines^1^. Four variants of the polled allele have been discovered^6^. These variants are referred to as Mongolian polled (P_M_)^7^, Guarani polled (P_G_)^8^, Friesian polled (P_F_)^9–11^, and Celtic polled (P_C_)^9,10^. This study focused on the P_C_ variant which is found in northern European beef breeds, such as Angus, and consists of the deletion of a 10 base pair (bp) segment replaced by a 212 bp duplication (Supplementary Fig. S1)^6,7,9^. There is no known transcript or protein associated with any polled variant, and the underlying mechanism resulting in the polled phenotype is not well understood.

Carlson et al. demonstrated that recapitulating the deletion and duplication of the P_C_ allele is sufficient to result in the polled phenotype. This was done by substituting the P_C_ allele for the p allele at the polled locus in a cell lined derived from a horned dairy bull, followed by somatic cell nuclear transfer (SCNT) cloning to produce two polled dairy bull calves^3^. A more recent study by Young et al. demonstrated that the P_C_ allele from the genome edited bull created by Carlson et al. is heritable^12^. Although successful introgression of the P_C_ allele was achieved and calves were produced, the overall efficiency of this process was low. Due to complications associated with cloning, three of the five calves were not viable and needed to be euthanized within 24 h of birth^3^. Only two calves survived to 60 days after birth; an overall efficiency of 7%^3^. Using a CRISPR-Cas12a gene editing approach, Schuster et al. also demonstrated the P_C_ allele is sufficient to result in the polled phenotype, however they too struggled with the inefficiencies of SCNT^13^. Low efficiency for producing calves via SCNT is not uncommon^14^. Due to low success, cloning would be an inefficient way to successfully integrate the Pc allele into elite genetic lines of horned cattle. An alternative option would be to edit the genome through direct injection of zygotes. Methods for producing in vitro fertilized (IVF) embryos have resulted in higher pregnancy rates^15–17^. Live, healthy genome edited animals produced through direct injection of bovine zygotes with either TALENs or CRISPRs have been reported at more efficient rates as well^18–20^.

The previously described studies showed that the P_C_ allele was sufficient to induce the polled phenotype, however it is not clear whether the 212 bp duplication, the 10 bp deletion, or both are necessary to result in the polled phenotype. In this study, we directly tested whether the deletion of the 10 bp segment alone can result in the polled phenotype by creating a targeted deletion in genotypically horned embryos to see if a polled phenotype would occur. Here, we also addressed the inefficiency of SCNT by directly editing zygotes using the CRISPR-Cas9 gene editing system. A dual guide RNA (gRNA) deletion approach was taken to delete a 133 bp region including the 10 bp missing at the polled locus (Supplementary Fig. S2). Timing of zygote microinjection and type of gRNA (synthetic or in vitro transcribed) were analyzed to optimize deletion efficiency. Once optimized, embryo transfers of presumptive 133 bp deletion embryos were performed, and the resulting fetuses were analyzed to determine if the deletion alone was sufficient to result in the polled phenotype.

## Results

### Guide-RNA testing

**Figure 1:**
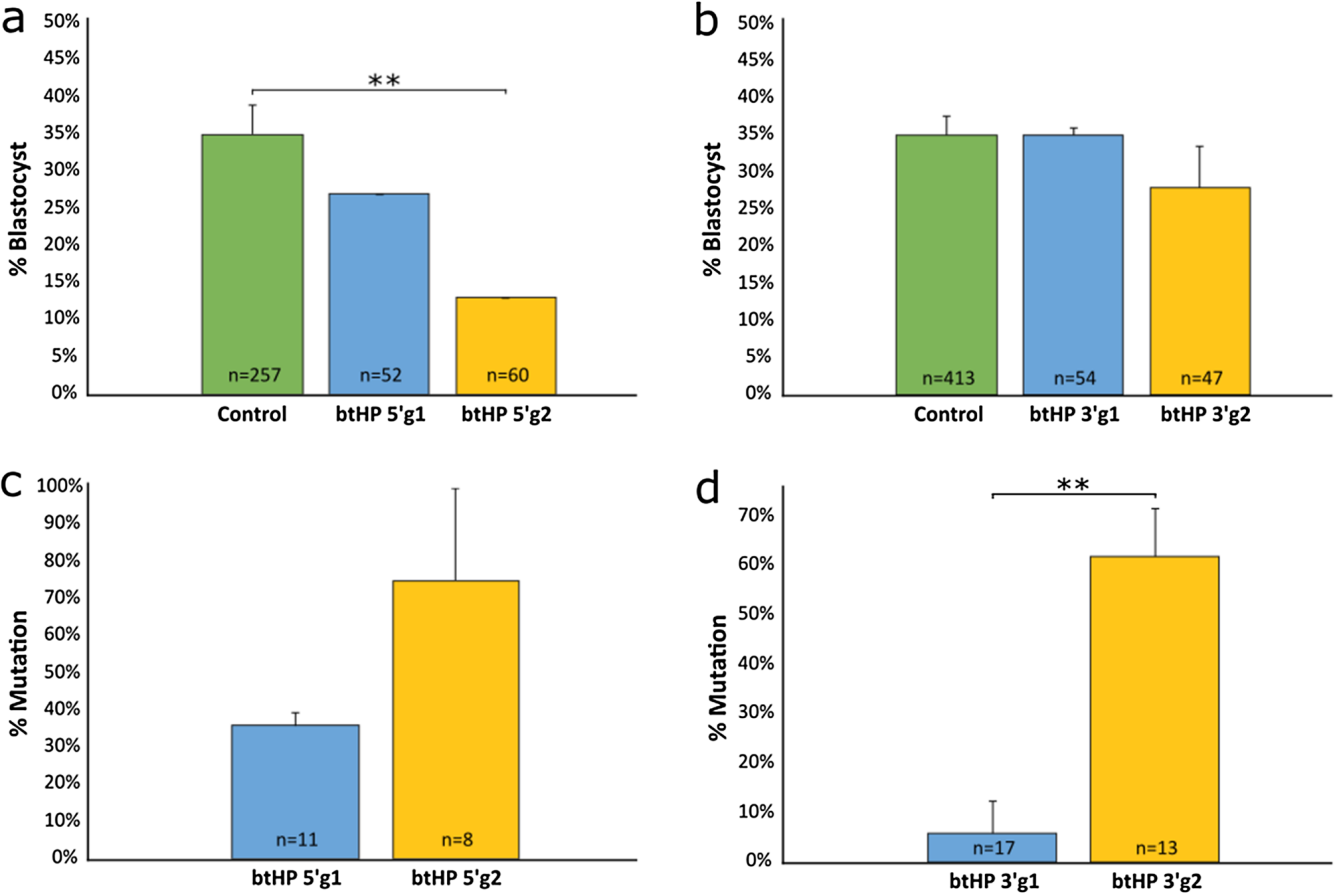
Non-injected and microinjected zygote development rates to the blastocyst stage and mutation efficiencies following microinjection of Cas9 protein and in vitro transcribed (IVT) test gRNAs targeting the 5′ and 3′ regions flanking the 10 bp on the polled locus at 18 h post insemination. (**a**) Blastocyst development rate of microinjected and non-injected control embryos (green) when targeting the 5′ and (**b**) 3′ regions flanking the 10 bp target region. (**c**) Mutation rates in embryos injected with Cas9 and gRNAs targeting the 5′ and (**d**) 3′ regions flanking the 10 bp target region. Error bars = SEM. **P < 0.01.

To optimize production of a 133 bp deletion in the bovine genome, gRNAs were designed targeting the 5′ and 3′ ends flanking the 10 bp sequence that is present in the horned allele but deleted in the P_C_ allele at the polled locus on bovine chromosome 1 (Supplementary Table S1; Supplementary Fig. S2). The top two gRNAs targeting the 5′ region (btHP 5′g1 and btHP 5′g2) and 3′ region (btHP 3′g1 and btHP 3′g2) were in vitro transcribed, incubated with Cas9 protein to form a ribonucleoprotein (RNP) complex and independently microinjected into zygotes 18 h post insemination (hpi). Groups of non-injected embryos were also cultured and used as developmental controls. The blastocyst rates for btHP 5′g1 and btHP 5′g2 were 27% and 13%, respectively (Fig. 1a; Supplementary Table S2). Non-injected controls developed at a rate of 35%, which was significantly higher than embryos microinjected with btHP 5′g2 (*P* = 0.005), but not btHP 5′g1 (*P* = 0.49). The development between the two microinjected groups was not significantly different (*P* = 0.17). btHP 5′g2 was chosen to be the 5′ guide due to its higher mutation rate (75%) compared to btHP 5′g1 (36%), though the difference was not statistically significant (Fig. 1c; Supplementary Table S2; *P* = 0.1). Of the 3′ target gRNAs, there was no significant difference in development between embryos microinjected with btHP 3′g1 (35%), btHP 3′g2 (28%) and non-injected controls (35%) (Fig. 1b; Supplementary Table S2). Embryos microinjected with btHP 3′g2 had a mutation rate of 62% which was significantly higher than the mutation rate of embryos microinjected with btHP 3′g1 (6%; *P* = 0.006) (Fig. 1d; Supplementary Table S2). Based on these results, btHP 3′g2 was used for the 3′ gRNA, which would result in a 133 bp deletion when microinjected in tandem with btHP 5′g2 (Supplementary Fig. S3).

### Timing of guide-RNA Co-injection and deletion efficiency

**Figure 2:**
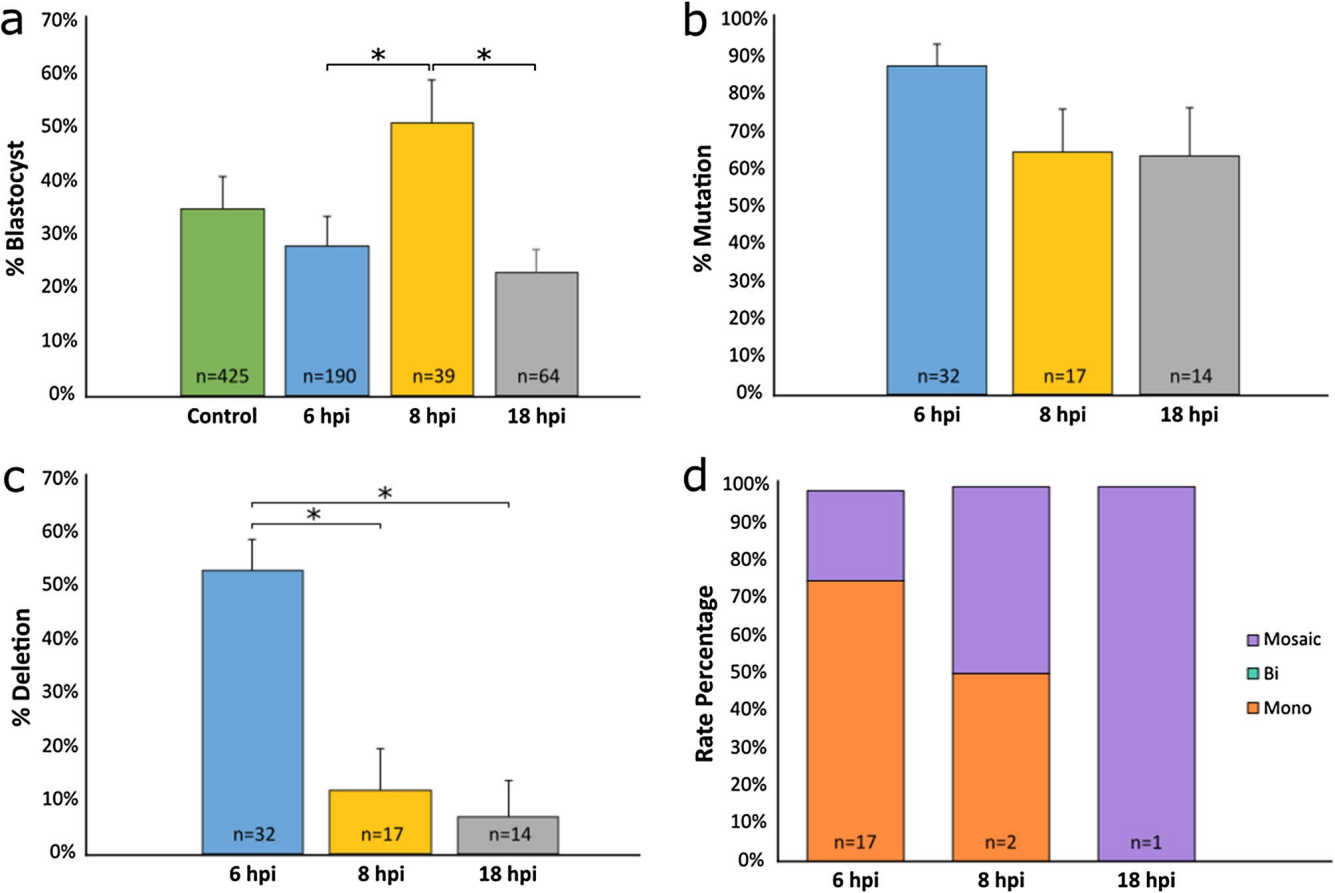
Non-injected and microinjected zygote blastocyst development rates and mutation and deletion efficiencies following microinjection of Cas9 protein and in vitro transcribed (IVT) gRNAs btHP 5′g2 and btHP 3′g2 at 6, 8 or 18 h post insemination (hpi). (**a**) Blastocyst development rate of microinjected and non-injected control embryos (green). (**b**) Mutation rates and (**c**) deletion rates in embryos injected with Cas9 protein, btHP 5′g2 and btHP 3′g2. A blastocyst was considered mutated if a mutation was detected at one or both target sites. (**d**) Frequency of types of deletions detected in microinjected embryos. Mono = monoallelic (orange); Bi = biallelic (aqua), Mosaic (purple). Error bars = SEM. *P < 0.05.

To test if the timing of the co-injection of the btHP 5′g2 and btHP 3′g2 RNP complexes affected deletion efficiency, zygotes were divided into three groups, and three trials of co-injections were done at 6, 8 or 18 hpi. The blastocyst rate of embryos microinjected 8 hpi (51%) was significantly higher than embryos microinjected 6 (28%; *P* = 0.025) and 18 hpi (and 23%; *P* = 0.022). Non-injected control embryos had a blastocyst rate of 35% (Fig. 2a; Supplementary Table S3). An embryo was considered positive for a mutation if a mutation was detected at either or both gRNA target sites. The individual mutation rates for each microinjected group were not significantly different (Fig. 2b; Supplementary Table S3), however the targeted 133 bp deletion rate, when both gRNAs cut in tandem, for the 6 hpi microinjected group (53%) was significantly higher when compared to either the 8 hpi (12%, *P* = 0.026) and 18 hpi (7%, *P* = 0.036) groups (Fig. 2c; Supplementary Table S3). An embryo was classified as mosaic if more than two alleles were detected. Of the embryos that had deletions, embryos microinjected 6 hpi had the lowest rate of mosaicism (24%) and only monoallelic deletions were detected (76%) (Fig. 2d; Supplementary Table S3). One embryo had a monoallelic deletion and one had a mosaic deletion in the 8 hpi microinjected group, and only one embryo with a deletion was detected in the 18 hpi group, and it was mosaic. Due to small sample sizes, it was not possible to detect a significant difference in the types of deletions when comparing times of injections.

### In vitro transcribed versus synthetic guide-RNA efficiency

**Figure 3:**
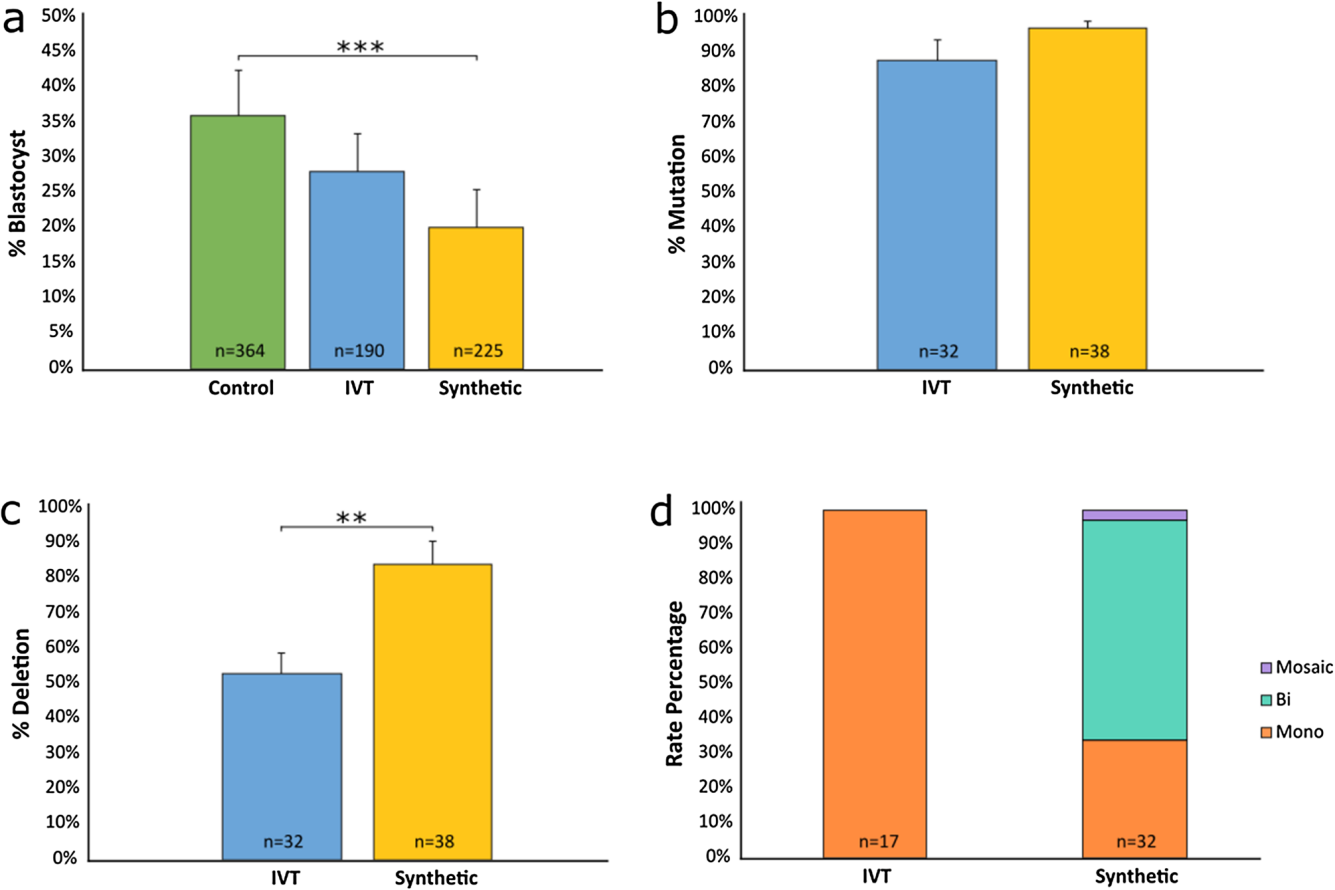
Non-injected and microinjected zygote blastocyst development rates and mutation and deletion efficiencies following microinjection of Cas9 protein and either in vitro transcribed (IVT) or synthetic gRNAs btHP 5′g2 and btHP 3′g2 at 6 h post insemination. (**a**) Blastocyst development rate of microinjected and non-injected control embryos (green). (**b**) Mutation rates and (**c**) deletion rates in embryos injected with Cas9 protein, btHP 5′g2 and btHP 3′g2. A blastocyst was considered mutated if a mutation was detected at one or both target sites. (**d**) Frequency of types of deletions detected in microinjected embryos. Mono = monoallelic (orange); Bi = biallelic (aqua), Mosaic (purple). Error bars = SEM. **P < 0.01; ***P < 0.001.

To determine if deletion efficiency could be further improved, a comparison of in vitro transcribed (IVT) and synthetic gRNAs was performed. Embryos were divided into two groups and microinjected in three trials at 6 hpi with RNP complexes using either IVT or synthetic btHP 5′g2 and btHP 3′g2 guides. The IVT gRNA group had a development rate of 28% while the synthetic gRNA group developed at a rate of 20%. Control non-injected embryos had a development rate of 36% (Fig. 3a; Supplementary Table S4). There was a significant difference in development rates between the non-injected controls and the synthetic gRNA group (*P* = 0.001), but there was no difference in development between the IVT gRNA group and non-injected controls (*P* = 0.151) or the IVT and synthetic gRNA microinjected group (*P* = 0.143). The mutation rate of embryos injected with either IVT or synthetic gRNAs was not significantly different (88% and 97%, respectively; *P* = 0.146) (Fig. 3b; Supplementary Table S4), but there was a significant difference in 133 bp deletion rates (*P* = 0.006)—embryos injected with IVT gRNAs had a 53% deletion rate while synthetic gRNA injected embryos had an 84% deletion rate (Fig. 3c; Supplementary Table S4). Of the embryos that had deletions, there were significant differences in monoallelic and biallelic deletions between IVT and synthetic gRNA injected embryos, with 100% of the IVT gRNA injected embryos having monoallelic deletions compared to 34% of synthetic gRNA injected embryos (*P* = 3.9 × 10^–7^). Furthermore, 63% of synthetic gRNA injected embryos had biallelic deletions while none were seen in IVT gRNA injected embryos (*P* = 1 × 10^–6^) (Fig. 3d; Supplementary Table S4). There was no significant difference in mosaicism between either gRNA types.

### Embryo transfers

In total, 78 day-7 presumptive 133 bp deletion embryos were transferred into 28 synchronized recipients—42 IVT gRNA edited embryos into 14 recipients and 36 synthetic gRNA edited embryos into 14 recipients (Table 1). Seven of the 14 recipients that received IVT gRNA edited embryos were pregnant at day 35 of gestation (50% pregnancy rate), with 12, possibly 13, fetuses detected (29–31% fetal development rate), but at 80 days of gestation, a total of three recipients were pregnant (21% pregnancy rate) with five fetuses detected (12% fetal development rate). Due to the high deletion rates we discovered using synthetic gRNAs, two embryo transfers were done using synthetic gRNA edited embryos. Only one of the 14 recipients that received synthetic gRNA edited embryos was pregnant at 35 and 80 days of gestation (7% pregnancy rate) with three fetuses (8% fetal development rate) detected.

**Table 1:** Results from embryo transfers (ETs) of zygotes injected 6 h post insemination with in vitro transcribed (IVT) or synthetic gRNAs and Cas9 protein.

ET	gRNA type	Blastocysts transferred	Recipients	35 days of gestation		80 days of gestation		Fetuses harvested (%)
				Pregnant (%)	Fetuses detected (%)	Pregnant (%)	Fetuses detected (%)	
1	IVT	18	6	2 (33)	4 (22)	2 (33)	4 (22)	3 (17)
2	IVT	24	8	5 (63)	8–9 (33–38)	1 (13)	1 (4)	1 (4)
Total	IVT	42	14	7 (50)	12–13 (29–31)	3 (21)	5 (12)	4 (10)
3	Synthetic	12	4	0 (0)	0 (0)	–	–	–
4	Synthetic	24	10	1 (10)	3 (8)	1 (7)	3 (13)	3 (13)
Total	Synthetic	36	14	1 (7)	3 (8)	1 (7)	3 (8)	3 (8)
Overall total	IVT & Synthetic	78	28	8 (29)	15–16 (19–21)	4 (14)	8 (10)	7 (9)

Two to three blastocysts were transferred per recipient. Pregnancies were confirmed on 35 and 80 days of gestation.

### Phenotypic and genotypic analysis of fetuses

**Figure 4:**
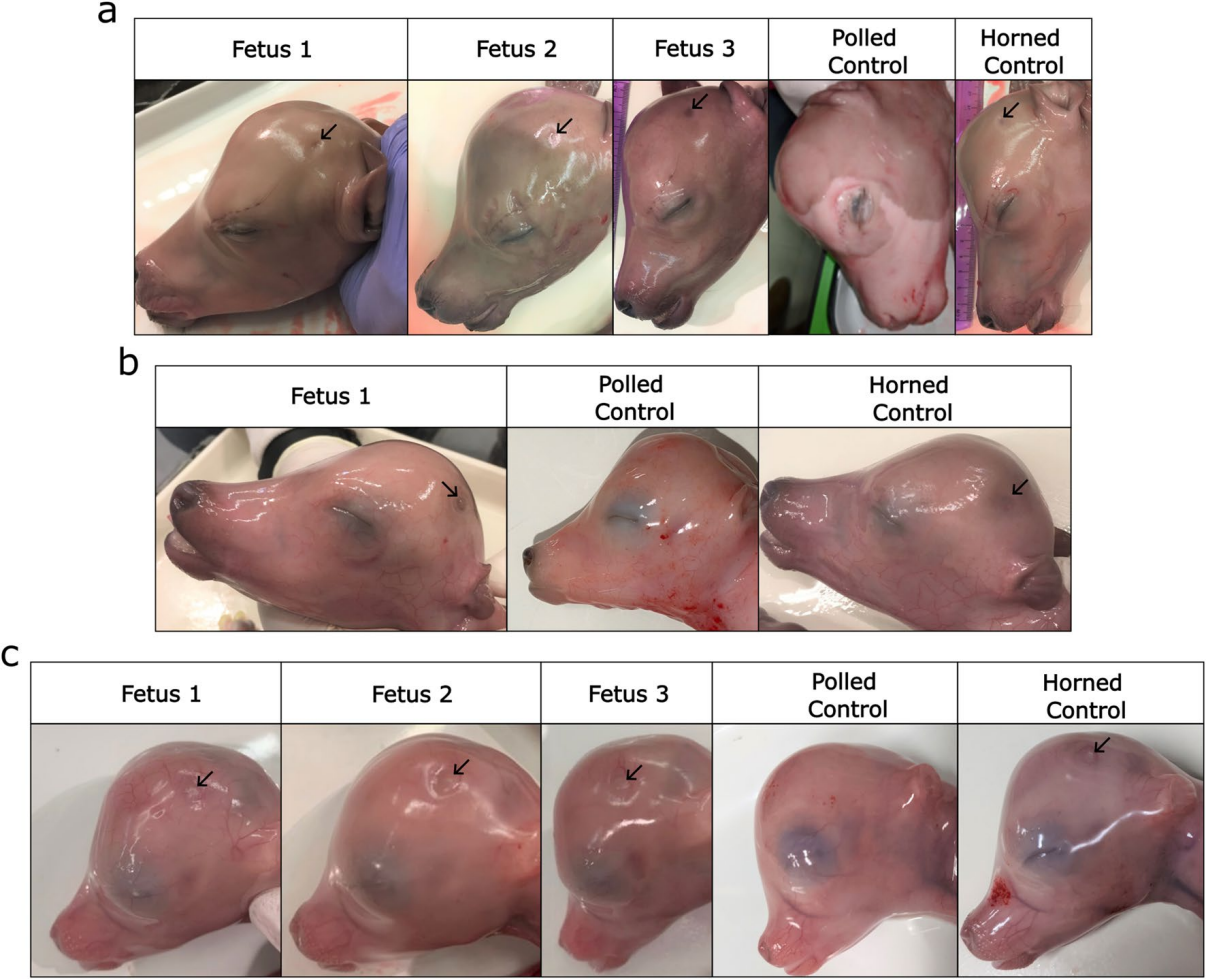
Phenotypic analysis of fetuses harvested from embryo transfers (ETs). (**a**) Fetuses from ET 1, (**b**) 2 and (**c**) 4 along with aged matched horned and polled controls. Fetuses were harvested at 151, 123 and 95 days of gestation, respectively. Horn bud development can be seen by the presence of small dimples (black arrows).

**Figure 5:**
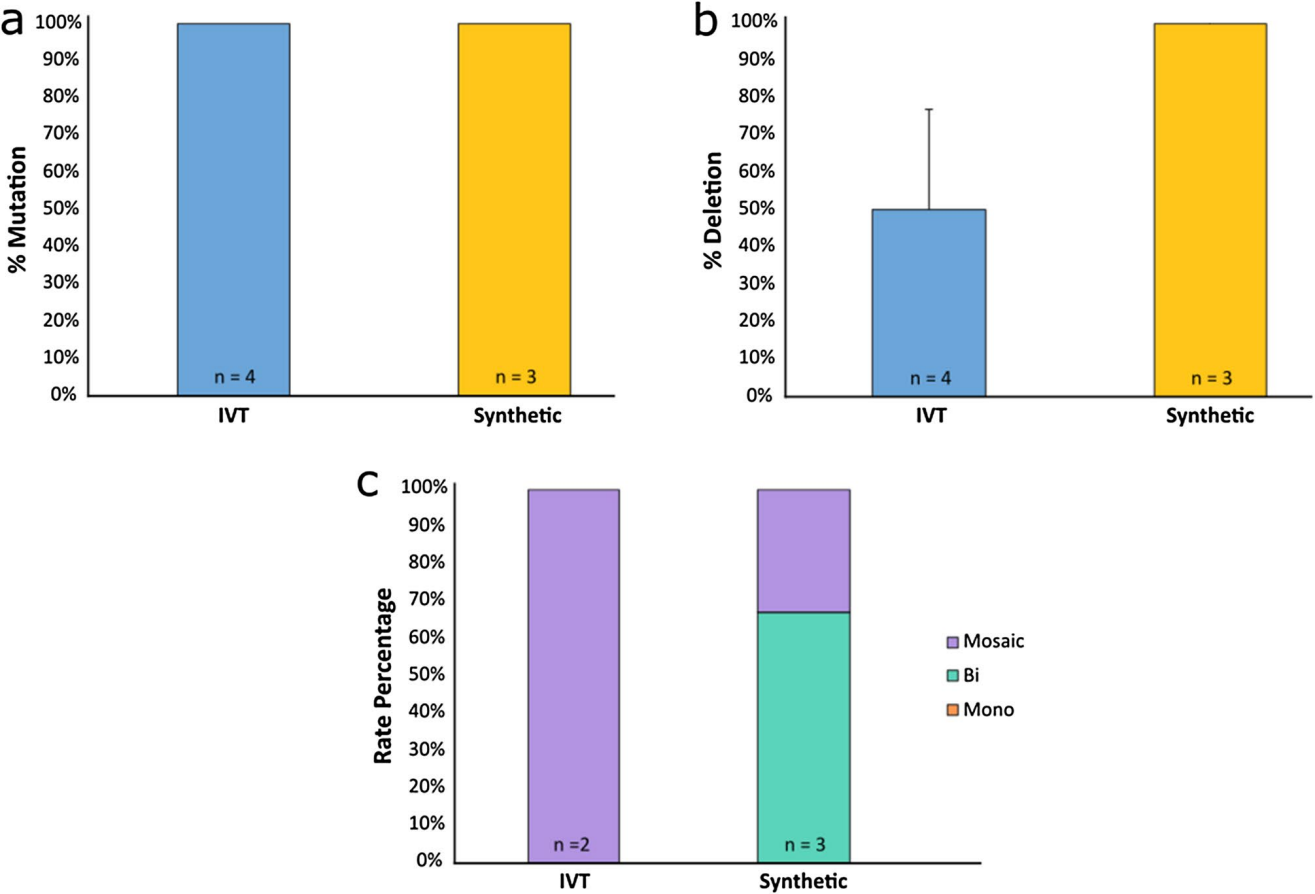
Mutation and deletion efficiencies of fetuses harvested from in vitro transcribed (IVT) and synthetic gRNA edited embryo transfers. (**a**) Mutation rates and (**b**) deletion rates in fetuses edited with Cas9 protein and either IVT or synthetic gRNAs btHP 5′g2 and btHP 3′g2. A fetus was considered mutated if a mutation was detected at one or both target sites. (**c**) Frequency of types of deletions detected in edited fetuses. Mono = monoallelic (orange); Bi = biallelic (aqua), Mosaic (purple). Error bars = SEM.

**Figure 6:**
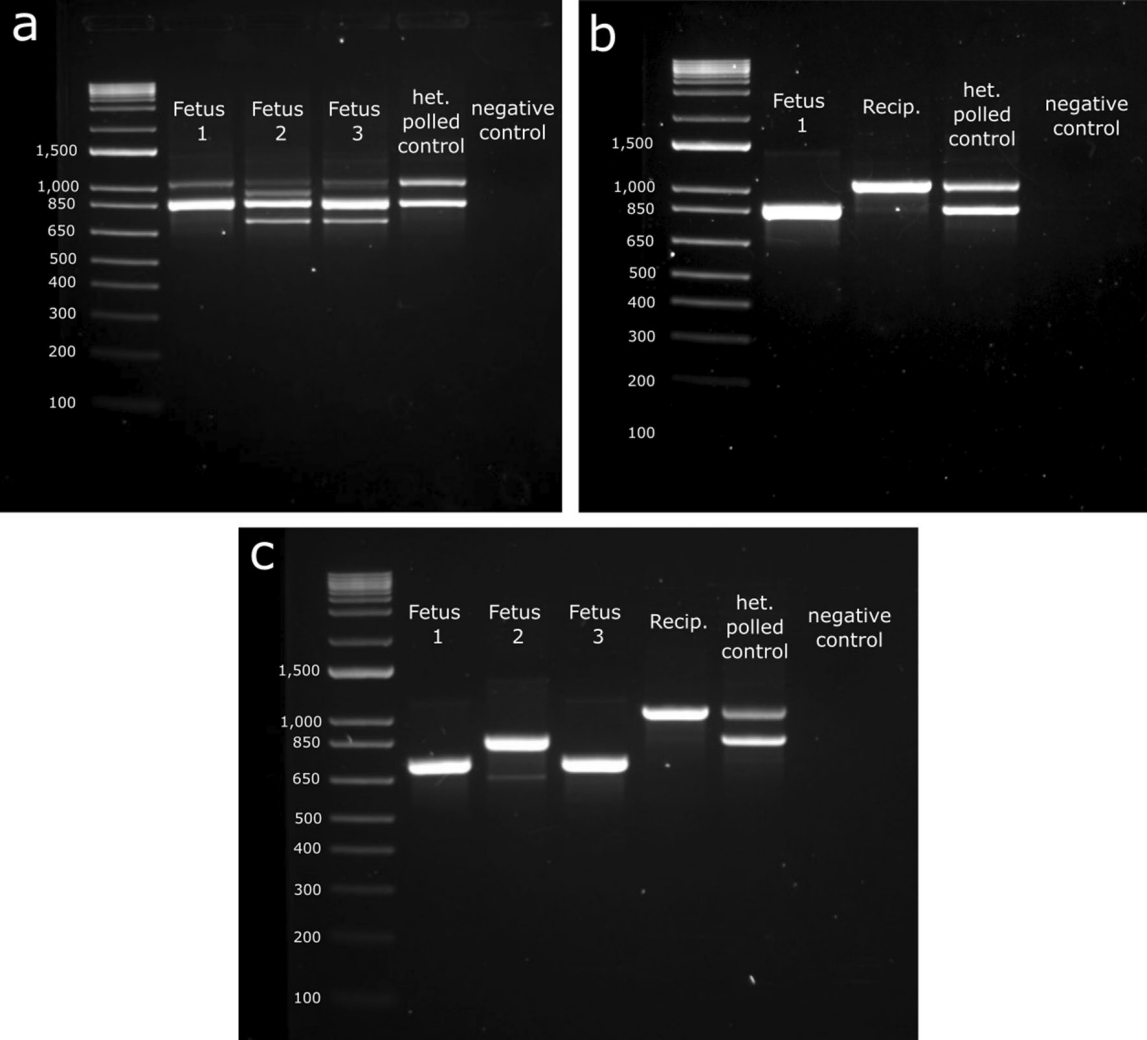
Genotypic analysis of fetuses harvested from embryo transfers (ETs). (**a**) Gel showing fetal genotypes from ET 1, (**b**) 2 and (**c**) 4. DNA was extracted from tail tissue and PCR amplified. Gel electrophoresis was done to visualize the 133 bp targeted deletion. Polled amplicon is 1078 bp, horned amplicon is 866 bp and expected size with deletion is 733 bp. Polled maternal DNA contamination can be seen in fetuses from ET1 (1078 bp bands).

Overall, a total of four IVT gRNA edited fetuses were harvested—three at 151 days of gestation and one at 123 days of gestation—(overall 10% fetal development rate), and three synthetic gRNA edited fetuses were harvested at 95 days of gestation (overall 8% fetal development rate) (Table 1). All fetuses presented small dimples in the horn bud region, indicating horn bud development had occurred (Fig. 4a–c). PCR and Sanger sequencing revealed that all had mutations at one or both target sites (Fig. 5a; Supplementary Table S5). Only two of the four IVT gRNA edited fetuses contained the 133 bp deletion, while all three synthetic gRNA edited fetuses had the deletion (Figs. 5b, 6a–c; Supplementary Table S5). Both IVT gRNA edited fetuses that had the deletion were mosaic, while only one synthetic gRNA edited fetus was mosaic, with the other two having biallelic deletions and no mosaicism detected (Fig. 5c; Supplementary Table S5). Due to small sample sizes, it was not possible to detect significant differences in the frequency of deletions or mosaicism rates between IVT or synthetic gRNA edited fetuses.

The polled (P_C_) allele was detected in all three fetal PCRs for ET1 (Fig. 6a), despite the fetuses clearly presenting a horned phenotype. It was deduced that there was maternal DNA contamination, as the recipients were polled Angus (positive for the P_C_ allele). Extra precaution and more thorough sample washes were done for the subsequent fetal harvests to avoid future maternal DNA contamination. Recipient DNA was taken for ETs 2 and 4 as a control due to the maternal DNA contamination issues from ET1. The P_C_ allele was only detected in the polled Angus recipient and no longer detected in the fetuses, demonstrating the modified sample processing protocol was effective (Fig. 6b,c). All fetuses were also tested for the P_F_ allele and all were negative.

### Histological analysis of fetuses

**Figure 7:**
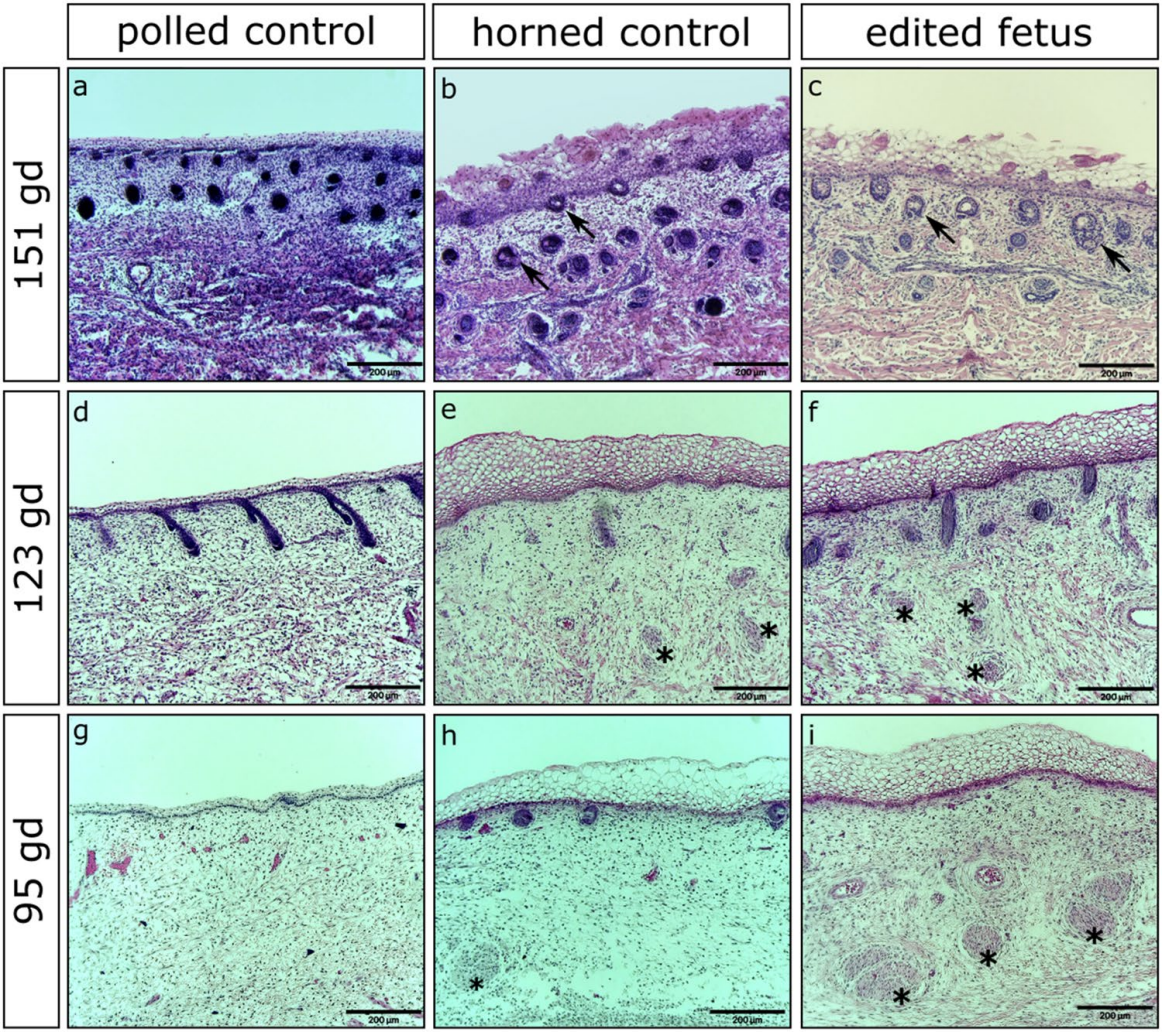
Histological analysis of gene edited fetuses along with polled and horned controls. (**a,d,g**) Horn bud region of aged matched polled and (**b**,**e**,**h**) horned control fetuses alongside representative fetuses from (**c**) ETs 1, (**f**) 2 and (**i**) 4. Multiple layers of vacuolated keratinocytes can be seen in the horn bud region of horned fetuses along with sebaceous glands (black arrows) and nerve bundles (black stars). Stained with Hematoxylin and eosin. gd = gestation days.

Histological analysis of fetuses from ET1, ET2 and ET4 revealed that all fetuses had horn bud development consistent with wild type horned control fetuses in that the horn bud region had several structural differences compared to their respective frontal skin (Supplementary Figs. S4–S10). Extensive layering of vacuolated keratinocytes was seen in the horn bud region of edited fetuses (Fig. 7c,f,i) as well as horned controls (Fig. 7b,e,h). The layering of the vacuolated keratinocytes in the frontal skin of all fetuses and the horn bud region of polled controls was not as prominent. Also like the horned controls (Fig. 7e,h), nerve bundles were seen in the horn bud region of edited fetuses (Fig. 7f,i), with little to no hair follicles present (Supplementary Figs. S7–S10). The frontal skin of all fetuses (Supplementary Figs. S7–S10) and the horn bud region of polled controls (Fig. 7d,g; Supplementary Figs. S7–S10) lacked nerve bundles and many hair follicles were present. In fetuses harvested at 151 days of gestation, sebaceous gland development was seen in the horn bud region of horned control fetuses (Fig. 7b) and edited fetuses (Fig. 7c), but was lacking in the frontal skin of all fetuses as well as the horn bud region of polled control fetuses (Fig. 7a; Supplementary Figs. S4–S6).

## Discussion

The findings from this study demonstrated that CRISPR-Cas9 dual (synthetic) guide RNAs microinjected as RNPs 6 hpi in bovine embryos is an efficient method to obtain biallelic deletion animals, however the 133 bp deletion, including the10 bp deletion found in the P_C_ allele, is not sufficient to result in the polled phenotype.

We designed gRNAs targeting the 5′ and 3′ regions surrounding the targeted 10 bp in genotypically horned embryos that resulted in high rates of mutation and, when co-injected, resulted in the predicted 133 bp deletion, the smallest possible deletion containing the 10 bp we could achieve based on gRNA design and mutation efficiency in embryos. We found that embryos microinjected 6 hpi with IVT gRNAs had much higher rates of deletion (53%) compared to those injected 8 or 18 hpi (12% and 7%, respectively) (Fig. 2c; Supplementary Table S3). Due to the high mutation efficiency when microinjecting embryos 6 hpi, this protocol was used for subsequent studies conducted in our lab^21–23^. It was interesting to note that the development rate for embryos microinjected 8 hpi was significantly higher compared to embryos injected 6 or 18 hpi (Fig. 2a; Supplementary Table S3). This could potentially be attributed to two factors: concentration of sperm used in IVF and higher editing efficiency. A concentration of 2 × 10^6^ sperm per mL was used for embryos microinjected 6 or 8 hpi, while a concentration of only 1 × 10^6^ sperm per mL was used for embryos microinjected 18 hpi. The sperm concentration was increased for embryos that were microinjected 6 or 8 hpi to help compensate for the shorter IVF incubation period. This increase in sperm concentration may have played a role in the higher blastocyst rate between embryos injected 8 hpi and 18 hpi. Embryos injected 6 hpi may have had a lower development rate because of the higher mutation and deletion rates compared to embryos microinjected 8 hpi. Our previous study^24^ demonstrated an inverse correlation between development and mutation rates generally occurs, with more efficient gRNAs resulting in lower blastocyst development rates. A trend was also seen among embryos that had deletions where the earlier the embryo was microinjected post-insemination, the lower the mosaicism rate became (Fig. 2d; Supplementary Table S3), but due to the small sample sizes in the 8 and 18 hpi groups, we were not able to detect a significant difference between the groups. This is consistent with the study by Lamas-Toranzo et al. where they microinjected gRNAs and Cas9 mRNA or RNP complexes into bovine embryos 0, 10 or 20 hpi and evaluated mosaicism levels. They found that the earlier the embryos were microinjected, the lower the mosaicism rate^25^. It is feasible that by introducing the editing reagents early, the genome can be edited before DNA replication occurs, resulting in a lower rate of mosaicism.

After synthetic gRNAs became available, we compared them with our IVT gRNAs to see if we could improve our deletion efficiency at 6 hpi. Interestingly, the synthetic gRNAs outperformed the IVT gRNAs in many respects. Although similar mutation efficiencies were seen between the two groups, a significantly higher number of embryos injected with synthetic gRNAs had the targeted 133 bp deletion (84%) compared to those injected with IVT gRNAs (53%) (Fig. 3c; Supplementary Table S4). Furthermore, of the embryos that had deletions, the majority of those injected with synthetic gRNAs had biallelic deletions, whereas only monoallelic deletions were detected in IVT injected embryos (Fig. 3d; Supplementary Table S4). This could be due to several factors. One could be quality control of gRNAs. We did not have easy access to a mass spectrometer to accurately measure product purity of the IVT gRNAs we produced ourselves. Conversely, commercial production companies can provide this with their synthetic gRNA products, allowing for a purer and more reliable product. The proprietary scaffold of synthetic gRNAs may also influence editing efficiency by potentially having a stronger or more readily forming bond with the Cas9 protein. It should also be noted that IVT gRNAs have been shown to trigger an innate immune response, whereas synthetic gRNAs do not^26^. Although this immune response was demonstrated in primary cell lines, one may question if this could translate over into embryos. Little is known about the innate immune response in early embryos, but a recent study unveiled the existence of what can be described as the earliest observable innate immune response in the developing embryo^27^. They discovered that epithelial cells were able to detect, consume, and destroy defective cells, thus aiding in the embryo’s ability to survive. We did not observe a significant difference in lethality between the IVT and synthetic gRNA injected embryos, but is an area for future research.

Although our pregnancy and fetal development rates following transfer of edited embryos to surrogate dams are not as high as those routinely obtained in industry, they are still superior to those of SCNT embryos. A total of four embryo transfers were done, two using IVT gRNA edited embryos and two with synthetic gRNA embryos. Overall, a total of four out of 28 recipients became pregnant (14% pregnancy rate) and a total of seven fetuses out of the 78 transferred embryos were recovered (development rate of 9%) (Table 1).

The results from ET1 were consistent with expectations. The conception rate of in vivo produced embryos is around 50%, whereas IVF produced embryos is typically between 30 and 40%^28^. With this decrease in conception rate of uninjected IVF produced embryos, we predicted a similar or slightly lower conception rate with IVF produced gene edited embryos, which we saw for ET1 (pregnancy rate of 33%). The pregnancy rates of embryo transfers 2–4 were much lower, ranging from 0 to 13%. There are several possible explanations for the low rates we experienced. At the first pregnancy check for ET2, there was a 63% pregnancy rate and a 33–38% fetal development rate, however at the second ultrasound, all but one pregnancy was lost. During the period between the first and second ultrasound check, our area was heavily affected by the Camp Fire that started in Paradise, California, with the air quality index (AQI) being in the hazardous classification for several days. It is possible this could have caused extreme stress on the recipients, resulting in early pregnancy fetal losses. A similar phenomenon was seen in rhesus macaques at the California National Primate Research Center (CNPRC), only a mile away from where our recipients were housed. The rate of pregnancy loss in rhesus macaques exposed to the Camp Fire was almost double that of controls^29^. Although the miscarriage rate seen at the CNPRC (18%) was not as high as was seen in our recipients (50%), it is still possible the Camp Fire played a role in the fetal losses experienced in ET2.

No pregnancies were achieved from ET3. It is possible heat could have played a factor since the transfer was done in July with temperature highs between 32 and 38 °C. At this time of the year, we typically see a drop in the quality of embryos produced, but we proceeded due to the availability of recipient heifers. The fourth embryo transfer was scheduled later in the year when embryo quality improved.

Although we experienced lower pregnancy and fetal development rates with our IVF edited fetuses compared to normal IVF rates, the rates were still higher than those achieved with SCNT, and no phenotypic anomalies were seen. The percent of SCNT embryos that develop to term is typically 0.5–5%^16^ and developmental abnormalities are not uncommon^14^. It is possible that transferring 2–3 blastocysts per recipient played a role in lower pregnancy rates due to potential competition of space and resources, but taking into consideration cost, resources, reduction in experimental animal numbers and the early pregnancy termination timeline for fetal harvests, it was deemed an appropriate approach to obtain a greater number of fetuses.

All genotypically horned edited fetuses that contained the 133 bp deletion presented with a horned phenotype. Although unlikely, it is still feasible the absent 10 bp in the P_C_ allele is solely responsible for the polled phenotype. It is possible the 10 bp deletion could be important based on spatial architecture of the chromosomal DNA. It remains a possibility that since the 133 bp deletion we created was larger than the 10 bp deletion alone, it may have not altered the DNA in a similar manner, thus the polled phenotype did not occur.

The histological findings of our edited fetuses resembled those of horned control fetuses (Fig. 7). There were noticeable structural differences between the horn bud region of our edited fetuses and horned controls compared to the horn bud region of polled and the frontal skin of all fetuses. Among these differences were increases in layering of vacuolated keratinocytes in the horn bud region of edited and horned control fetuses as well as the presence of nerve bundles and sebaceous glands. These results are consistent with the histological findings seen in the work by others^9,30^. Thickening in the layering of vacuolated keratinocytes and the development of nerve bundles are the first notable differences in the differentiation of the horn bud region, occurring as early as 2–3 months of gestation^30^. The development of sebaceous glands in the horn bud region of horned fetuses occurs a few months later, approximately 4–5 months of gestation. Interestingly, the horn bud region of horned fetuses tends to differentiate before the forehead region as shown by a matured epidermis and the presence of sebaceous glands, with the maturation of the frontal skin being delayed about 1–2 months in comparison^30^.

Overall, this study reports the creation of an optimized CRISPR-Cas9 dual guide approach. We demonstrated that the time in which gene editing reagents are introduced into the zygote has a significant effect on deletion efficiency, and the use of synthetic gRNAs results in significantly higher deletion rates as well as lower levels of mosaicism compared to IVT gRNAs. Genotypically 133 bp biallelic deletion fetuses were obtained, but all displayed horn bud development, indicating that removal of the 133 bp encompassing the 10 bp DNA sequence typically present in the horned allele of the polled locus in genotypically horned (pp) embryos is not sufficient to result in the polled phenotype. Further research is needed to fully elucidate how the 212 bp duplication/10 bp deletion in the Celtic polled (P_C_) allele results in the dominant polled phenotype.

## Materials and methods

### Animal care

All experiments carried out utilizing animals were conducted in compliance with the ARRIVE guidelines and approved and completed in compliance with the Institutional Animal Care and Use Committee (IACUC) protocol #20746 at the University of California, Davis. Housing and maintenance of recipient cattle was conducted at the University of California, Davis Beef Barn and Feedlot.

### Guide-RNA design and construction

Guide sequences were designed using the online tools sgRNA Scorer 2.0^31,32^ and Cas-OFFinder^33^ targeting the 5′ and 3′ regions flanking the 10 bp target at the polled locus. Guides were selected with no less than three mismatches in the guide sequence for off-target sites using the UMD3.1.1 bovine reference genome^34^, and at least one mismatch in the seed region (8–11 bp upstream of the PAM sequence). Oligonucleotides were ordered from Eurofins USA (Louisville, KY) for the top four guides for construction of the gRNAs (two targeting upstream and two targeting downstream of the 10 bp target). In vitro transcription of the oligonucleotides was done using the AmpliScribe T7-Flash Transcription kit (Lucigen, Palo Alto, CA) and purified using the MEGAclear Transcription Clean-Up kit (Thermo Fisher, Chicago, IL) as described by Vilarino et al^35^. Synthetic guides targeting the same sequences were also ordered from Synthego (Menlo Park, CA) with the option of no modifications being done to the gRNAs. Cleavage efficiency was tested using an in vitro cleavage assay by combining 60 ng of PCR amplified product, 100 ng of gRNA, 150 ng of Cas9 protein (PNA Bio, Inc., Newbury Park, CA), 1 μL of 10X BSA, 1 μL of NEB Buffer 3.1 and water bringing the total volume to 10 μL in a 0.2 μL tube and incubated at 37 °C for 1 h. The incubated product was then run on a 2% agarose gel with 5 μL of SYBR Gold (Invitrogen, Waltham, MA) at 100 V for 1 h and visualized using a ChemiDoc-ItTS2 Imager (UVP, LLC, Upland, CA).

### Embryo production

Bovine ovaries were collected from a local processing plant and transported to the laboratory at 35–37 °C in sterile saline. Cumulus-oocyte complexes (COCs) were aspirated from follicles and groups of 50 COCs were transferred to 4-well dishes containing 500 μL of maturation media (BO-IVM, IVF Bioscience, Falmouth, United Kingdom). COCs were incubated for 20–22 h at 38.5 °C in a humidified 5% CO_2_ incubator. Approximately 25 oocytes per drop were fertilized in 60 μL drops of SOF-IVF with either 2 × 10^6^ sperm per mL for an incubation period of 6 or 8 h or 1 × 10^6^ sperm per mL for an incubation period of 18 h at 38.5 °C in a humidified 5% CO_2_ incubator using the protocol described in Bakhtari et al^36^. Sperm used was from a known genotypically horned sire. Previous preliminary experiments we conducted revealed the majority of ovaries obtain from the processing plant were from horned cattle, allowing for the creation of genotypically horned embryos. Presumptive zygotes were denuded by light vortex in SOF-HEPES medium^36^ for 5 min. 25 zygotes per drop were incubated in 50 μL drops of culture media (Bo-IVC, IVF Bioscience, Falmouth, United Kingdom) at 38.5 °C in a humidified atmosphere of 5% CO_2_, 5% O_2_ and 90% N_2_ for 7–8 days from insemination.

### Guide-RNA testing

Mutation rates for each guide were determined by laser-assisted cytoplasmic injection^37^ of in vitro fertilized embryos with 6pL of a solution containing 67 ng/μL of gRNA and 167 ng/μL of Cas9 protein (PNA Bio, Inc., Newbury Park, CA) incubated at room temperature for 30 min prior to injection to allow for the formation of RNP complexes. Injected embryos were incubated for 7–8 days from insemination. Embryos that reached blastocyst stage were lysed in 10 μL of Epicenter DNA extraction buffer (Lucigen, Palo Alto, CA) using a Simpli-Amp Thermal Cycler (Applied Biosystems, Foster City, CA) at 65 °C for 6 min, 98 °C for 2 min and held at 4 °C. The target region was amplified by two rounds of the polymerase chain reaction (PCR) using primers developed with Primer3 (Supplementary Table S6)^38,39^. The first round of PCR was performed on a SimpliAmp Thermal Cycler (Applied Biosystems, Foster City, CA) with 10 μL GoTAQ Green Master Mix (Promega Biosciences LLC, San Luis Obispo, CA), 0.4 μL of each primer at 10 mM and 9.2 μL of DNA in lysis buffer for 5 min at 95 °C, 35 cycles of 30 s at 95 °C, 30 s at 62 °C, and 2 min at 72 °C, followed by 5 min at 72 °C. The second round of PCR was run with 10 μL GoTAQ Green Master Mix (Promega Biosciences LLC, San Luis Obispo, CA), 4.2 μL of water, 0.4 μL of each primer at 10 mM and 5 μL of first round PCR for 3 min at 95 °C, 35 cycles of 30 s at 95 °C, 30 s at 60 °C, and 1 min at 72 °C, followed by 5 min at 72 °C. Products were visualized on a 1.3% agarose gel using a ChemiDoc-ItTS2 Imager (UVP, LLC, Upland, CA), purified using the QIAquick Gel Extraction Kit (Qiagen, Valencia, CA), Sanger sequenced (GENEWIZ, LLC, South Plainfield, NJ) and analyzed with CRISP-ID^40^ and Synthego’s ICE analysis. Mutation rates for co-injected in vitro fertilized embryos were determined using the same methods described above—injecting RNP complexes of the most efficient 5′ and 3′ IVT or synthetic gRNAs (67 ng/μL each) and 167 ng/μL of Cas9 protein (PNA Bio, Inc., Newbury Park, CA).

### Embryo transfers

Estrus synchronization of recipient cattle began 16 days prior to the embryo transfer with the use of an intravaginal progesterone releasing device (1.38 g; Eazi-Breed CIDR; Zoetis) and the administration of gonadorelin (100 mcg; Factrel; Zoetis) done on day 0. On day 7, the CIDR was removed and prostaglandin (25 mg; Lutalyse; Zoetis) was administered. A second dose of gonadorelin (100 mcg; Factrel; Zoetis) was given on day 9 and recipients were monitored for signs of estrus. Confirmation of recipient synchronization was done on day 15 via corpus luteum detection using a transrectal ultrasound. On day 16, embryo transfers were performed. Recipients received a caudal epidural of 100 mg 2% lidocaine (Xylocaine; Fresenius) prior to embryo transfer. Two to three blastocysts were loaded into 0.25 cc straws and transferred using the non-surgical transcervical technique into the uterine horn ipsilateral to the corpus luteum. On day 35 of gestation, transrectal ultrasonography (5.0 MHz linear probe; EVO Ibex, E.I. Medical Imaging) was done to confirm pregnancies, and reconfirmed on day 80. A total of four embryo transfers were performed, and recipients were resynchronized for subsequent embryo transfers if they did not become pregnant from prior embryo transfers.

### Phenotypic and genotypic analysis of fetuses

Recipient cattle were slaughtered between 95 and 151 days of gestation via penetrating captive bolt and subsequent exsanguination. The fetuses were retrieved from the uterine horns, and horn bud phenotyping was performed by the large animal veterinarian onsite. Fetal liver and tail samples were taken for later genotypic analysis, and the frontal skin and horn bud regions were taken for histological analysis. Recipient muscle tissue was also taken for experimental controls. All samples were washed three times in PBS before collection.

To determine fetal genotypes, DNA was extracted from tissue samples using the DNeasy Blood & Tissue Kit (Qiagen, Valencia, CA) and PCR amplified with the 2nd round primers used for gRNA testing. PCR was done using a SimpliAmp Thermal Cycler (Applied Biosystems, Foster City, CA) with 12.5 μL GoTAQ Green Master Mix (Promega Biosciences LLC, San Luis Obispo, CA), 1 μL of each primer at 10 mM, 9.5 μL of water and 100 ng of DNA for 5 min at 95 °C, 35 cycles of 30 s at 95 °C, 30 s at 60 °C, and 1 min at 72 °C, followed by 5 min at 72 °C. Products were visualized on a 1.3% agarose gel using a ChemiDoc-ItTS2 Imager (UVP, LLC, Upland, CA), purified using the QIAquick Gel Extraction Kit (Qiagen, Valencia, CA), Sanger sequenced (GENEWIZ, LLC, South Plainfield, NJ) and analyzed with CRISP-ID [44] and Synthego’s ICE analysis. Fetuses were also tested for the P_F_ allele using the same PCR protocol with a modified anneal and extension temp for the P_F_ primers given in Supplementary Table S6. The P_M_ and P_G_ alleles were not tested for as they were not applicable based on the breeds of cattle used to produce the fetuses.

### Histological analysis of fetuses

Fetal horn bud and frontal skin tissue samples were fixed in 4% paraformaldehyde at 4 °C for 18 h, washed 3 × in phosphate-buffered saline (PBS) on a rocker for 30 min and placed in 70% ethanol. They were subsequently processed in a vacuum infiltration processor (Sakura Tissue-Tek VIP 5, Torrance, CA) where they were dehydrated in a graded ethanol series and cleared with xylene. Samples were then embedded in paraffin blocks and 5 µm microtome sections were cut (Leica RM2255, Leica Biosystems, Buffalo Grove, IL) and stained with hematoxylin and eosin. Digital images were obtained with an Echo Revolve (Discover Echo Inc., San Diego, CA) microscope.

### Statistical analysis

Comparison between blastocyst and fetal development, mutation and deletion rates were evaluated using logistic regression models created with the glm “general linear model” function in R with gRNA, gRNA type, and time of injection modeled as fixed effects. Differences were considered significant when *P* < 0.05.

## Supplementary Information

Supplementary Information.

## Data Availability

Data generated and analyzed during this study are included in this published article and its Supplementary Information file.
