# Supplementary material for: A deletion at the polled PC locus alone is not sufficient to cause a polled phenotype in cattle
Source: Sci Rep. 2022 Feb 8;12:2067. doi: 10.1038/s41598-022-06118-6 (PMC8825853; doi:10.1038/s41598-022-06118-6)
Supplement: Supplementary file 1 — Supplementary Information. [file 41598_2022_6118_MOESM1_ESM.pdf]

Supplementary Data

A deletion at the polled P_C locus alone is not sufficient to cause a polled phenotype in cattle

Sadie L. Hennig[1], Joseph R. Owen[1], Jason C. Lin[1], Bret R. McNabb[2], Alison L. Van Eenennaam[1] and James D. Murray[1,2]

[1]Department of Animal Science, University of California – Davis, Davis, CA

[2]Department of Population Health and Reproduction, School of Veterinary Medicine, University of California – Davis, Davis, CA

Corresponding Author email: jdmurray@ucdavis.edu

SUPPLEMENTARY DATA

Supplementary Table S1. Guide RNA targets surrounding the 10 bp on the P_C allele. The location is given in respect to the 10 bp targeted deletion.

Location	Name	Sequence
5’	btHP 5’g1	GATAGTTTTCTTGGTAGGC
	btHP 5’g2	TCTTTGTAGTGAGAGCAGGC
3’	btHP 3’g1	GTCTATCCCAAAAGTGTGGG
	btHP 3’g2	GATGTTGAATTATAGGCAGA

Supplementary Table S2. Blastocyst and mutation rates of zygotes following microinjection of Cas9 protein and *in vitro* transcribed (IVT) test gRNAs targeting the 5’ and 3’ regions flanking the 10 bp on the polled locus. Bovine embryos were *in vitro* fertilized and microinjected 18 hours post insemination. Blastocysts were counted on day 8 and mutations were determine using Sanger sequencing. Letters that differ in the same column are significantly different ($P < 0.01$).

Target Region	gRNA	Total Embryos	Total Blastocysts (%)	Total Analyzed	Total Mutation (%)
5’	Non-Injected Control	257	90 (35)^{A}	-	-
	btHP 5’g1	52	14 (27)^{AB}	11	4 (36)^{A}
	btHP 5’g2	60	8 (13)^{B}	8	6 (75)^{A}
3’	Non-Injected Control	413	144 (35)^{A}	-	-
	btHP 3’g1	54	19 (35)^{A}	17	1 (6)^{A}
	btHP 3’g2	47	13 (28)^{A}	13	8 (62)^{B}

Supplementary Table S3. Blastocyst, mutation and targeted deletion rates of zygotes following microinjection of Cas9 protein and *in vitro* transcribed (IVT) gRNAs btHP 5'g2 and btHP 3'g2 targeting the polled locus. Bovine embryos were *in vitro* fertilized and microinjected 6, 8 or 18 hours post insemination (hpi). Blastocysts were counted on day 8 and mutations were determine using Sanger sequencing. A blastocyst was classified as mutated if a mutation was detected at either or both gRNA target sites. Total deletion rates are broken down into subsets of monoallelic (mono), biallelic (bi) and mosaic deletions. Letters that differ in the same column are significantly different ($P < 0.05$).

Time of Injection	Total Embryos	Total Blastocysts (%)	Total Analyzed	Total Mutation (%)	Total Deletion (%)	Subset of Deletion Embryos		
						Non-Mosaic		Mosaic
						Mono (%)	Bi (%)	
Non-Injected Control	425	148 (35)[ab]	-	-	-	-	-	-
6 hpi	190	53 (28)[b]	32	28 (88)[a]	17 (53)[a]	13 (76)	0 (0)	4 (24)
8 hpi	39	20 (51)[a]	17	11 (65)[a]	2 (12)[b]	1 (50)	0 (0)	1 (50)
18 hpi	64	15 (23)[b]	14	9 (64)[a]	1 (7)[b]	0 (0)	0 (0)	1 (100)

Supplementary Table S4. Blastocyst, mutation and targeted deletion rate of zygotes following microinjection of Cas9 protein and *in vitro* transcribed (IVT) or synthetic gRNAs btHP 5’g2 and btHP 3’g2 targeting the polled locus. Bovine embryos were *in vitro* fertilized and microinjected 6 hours post insemination (hpi). Blastocysts were counted on day 8 and mutations were determine using Sanger sequencing. A blastocyst was classified as mutated if a mutation was detected at either or both gRNA target sites. Total deletion rates are broken down into subsets of monoallelic (mono), biallelic (bi) and mosaic deletions. Letters that differ in the same column are significantly different, with capital letters at the 0.001 level ($P < 0.001$) and lowercase letters at the 0.01 level ($P < 0.01$).

gRNA Type	Total Embryos	Total Blastocysts (%)	Total Analyzed	Total Mutation (%)	Total Deletion (%)	Subset of Deletion Embryos		
						Non-Mosaic		Mosaic
						Mono (%)	Bi (%)	
Non-Injected Control	364	130 (36)^{A}	-	-	-	-	-	-
IVT	190	53 (28)^{AB}	32	28 (88)^{a}	17 (53)^{a}	17 (100)^{A}	0 (0)^{A}	0 (0)^{a}
Synthetic	225	45 (20)^{B}	38	37 (97)^{a}	32 (84)^{b}	11 (34)^{B}	20 (63)^{B}	1 (3)^{a}

Supplementary Table S5. Fetal genotyping results from embryo transfers (ETs) of zygotes injected 6 hr post insemination with *in vitro* transcribed (IVT) or synthetic gRNAs and Cas9 protein. A fetus was classified as mutated if a mutation was detected at either or both gRNA target sites. Total deletion rates are broken down into subsets of monoallelic (mono), biallelic (bi) and mosaic.

ET	gRNA Type	Days of Gestation	Total Fetuses	Total Mutation (%)	Total Deletion (%)	Subset of Deletion Fetuses		
						Non-Mosaic		Mosaic
						Mono (%)	Bi (%)	
1	IVT	151	3	3 (100)	2 (67)	0 (0)	0 (0)	2 (100)
2	IVT	123	1	1 (100)	0 (0)	-	-	-
**Total**	**IVT**	**-**	**4**	**4 (100)**	**2 (50)**	**0 (0)**	**0 (0)**	**2 (100)**
4	Synthetic	95	3	3 (100)	3 (100)	0 (0)	2 (67)	1 (33)
**Overall Total**	**IVT & Synthetic**	**-**	**7**	**7 (100)**	**5 (71)**	**0 (0)**	**2 (40)**	**3 (60)**

Supplementary Table S6. Primers used for PCR amplification of the P_C allele containing the targeted deletion and the P_F allele.

Target	PCR Round (1st or 2nd)	Forward	Reverse	Tm (°C)	Extension Time
P_C allele	1st	GGGCAAGTTGCTCAGCTGTTTTTG	TCCGCATGGTTTAGCAGGATTCA	62	2 min
	2nd	GAAGTGTGGCCGGTAGAAAA	TCCGCATGGTTTAGCAGGATTCA	60	1 min
P_F allele	1st	CCATCTTGGGTACAGCGTTT	TGTTCTGTGTGGGTTTGAGG	60	30 s

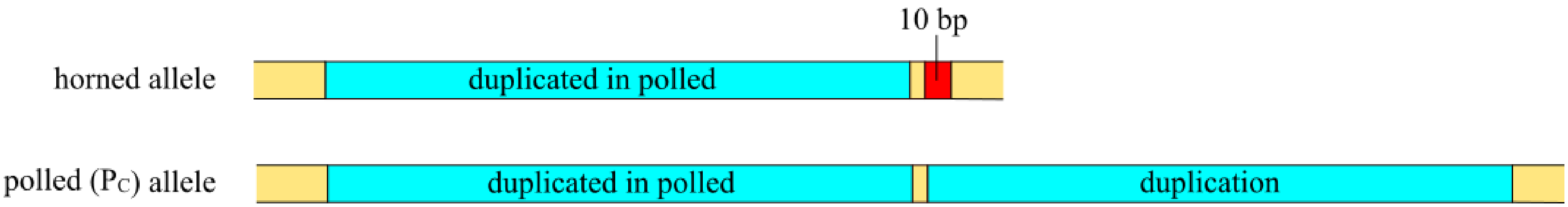

Supplementary Figure S1. Schematic representation of the horned versus the polled (P_C) allele.

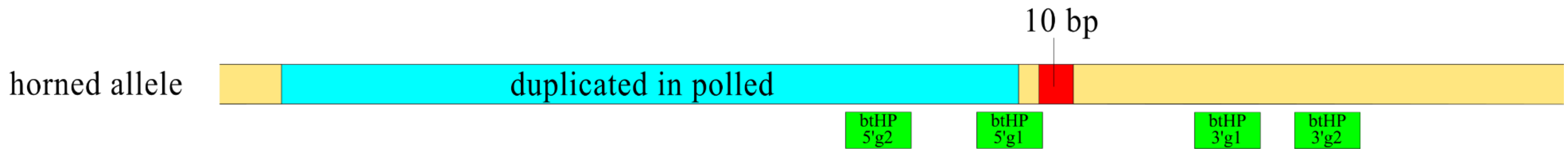

Supplementary Figure S2. Schematic representation of the designed gRNA locations targeting the 10 bp on the horned allele.

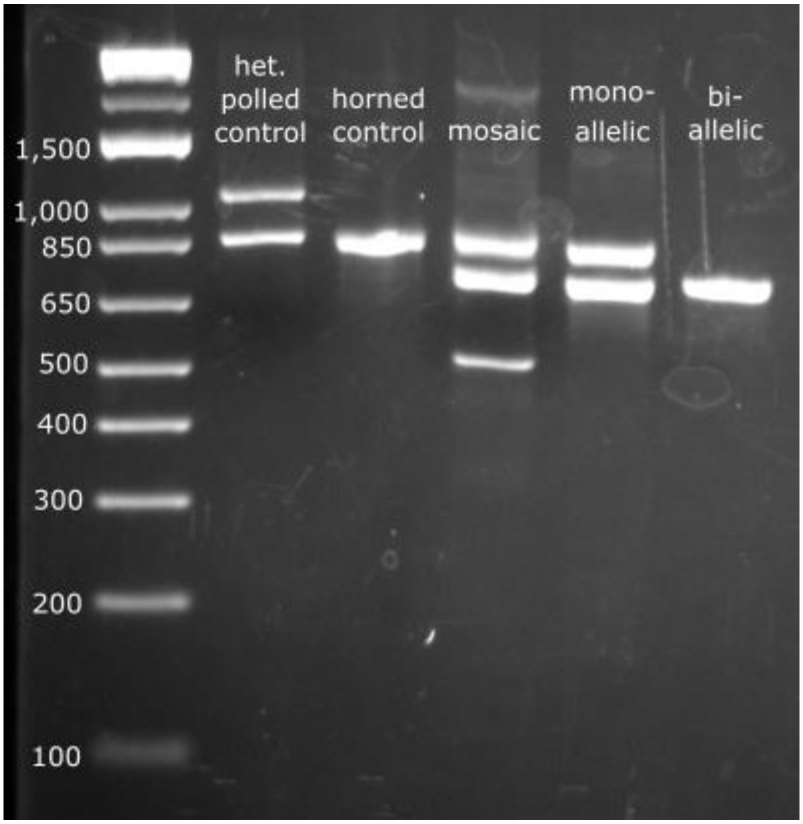

Supplementary Figure S3. Deletion detection at the polled locus of embryos injected with RNP complexes containing gRNAs btHP 5’g2 and btHP 3’g2. Following culture to day-8 blastocysts, DNA was extracted and gel electrophoresis was done. Polled amplicon is 1,078 bp, horned amplicon is 866 bp and expected size with deletion is 733 bp.

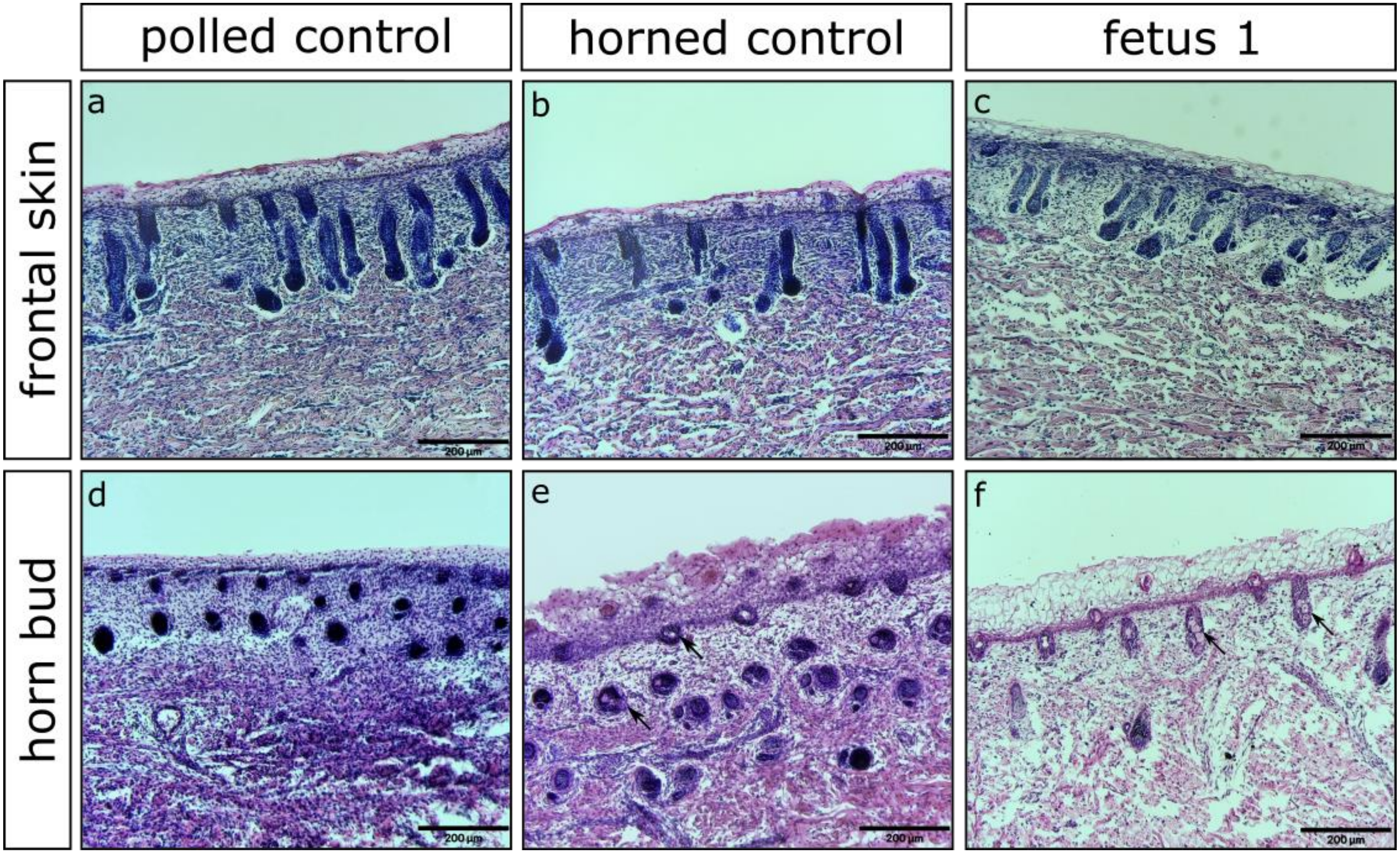

Supplementary Figure S4. Histological analysis of fetus 1 from embryo transfer 1 along with horned and polled control fetuses. (**a-c**) Frontal skin and (**d-f**) horn bud region of fetus 1 and age matched polled and horned controls at 151 days of gestation. Multiple layers of vacuolated keratinocytes can be seen in the horn bud region of horned fetuses along with sebaceous glands (black arrows). Stained with Hematoxylin and eosin.

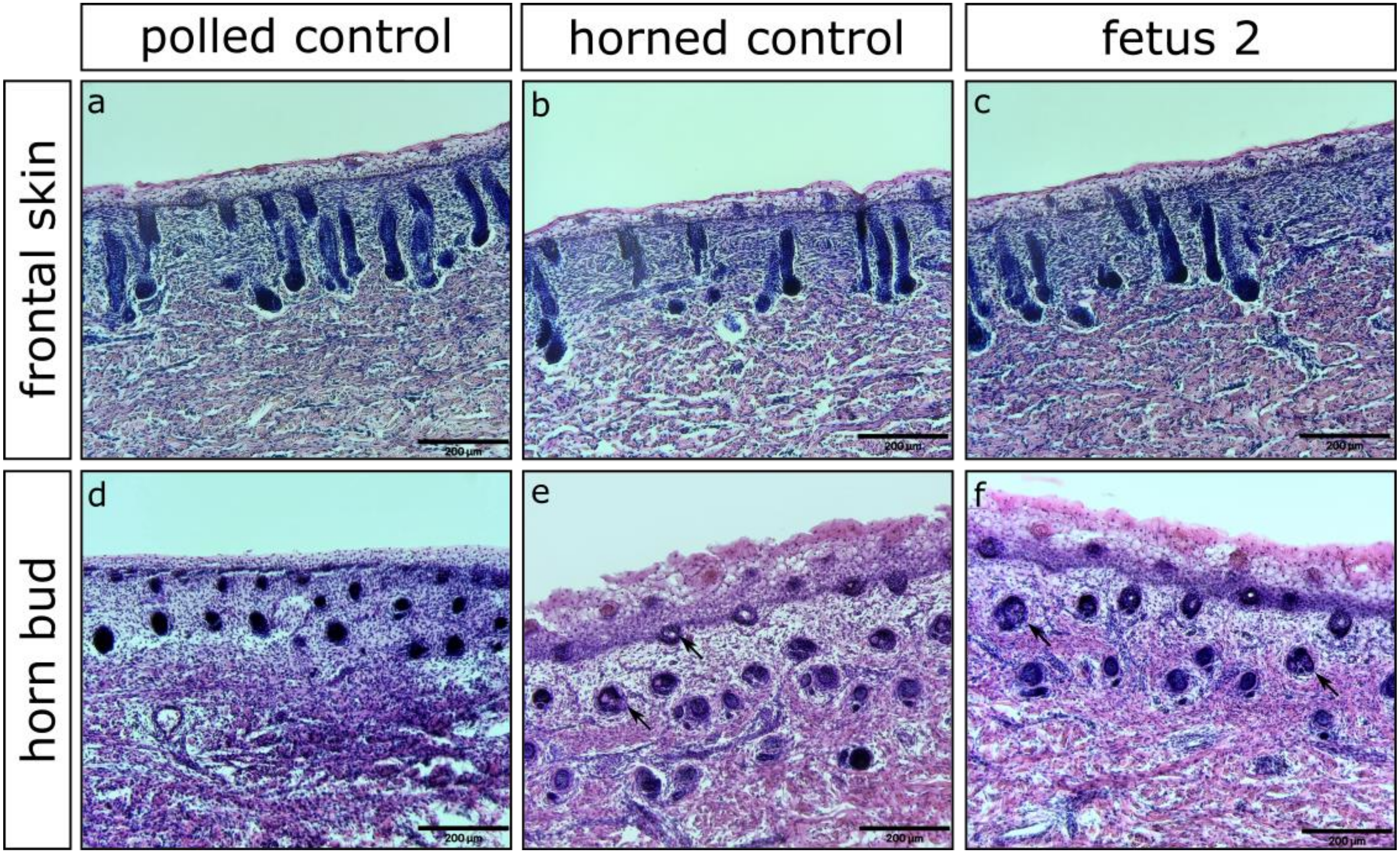

Supplementary Figure S5. Histological analysis of fetus 2 from embryo transfer 1 along with horned and polled control fetuses. (**a-c**) Frontal skin and (**d-f**) horn bud region of fetus 2 and age matched polled and horned controls at 151 days of gestation. Multiple layers of vacuolated keratinocytes can be seen in the horn bud region of horned fetuses along with sebaceous glands (black arrows). Stained with Hematoxylin and eosin.

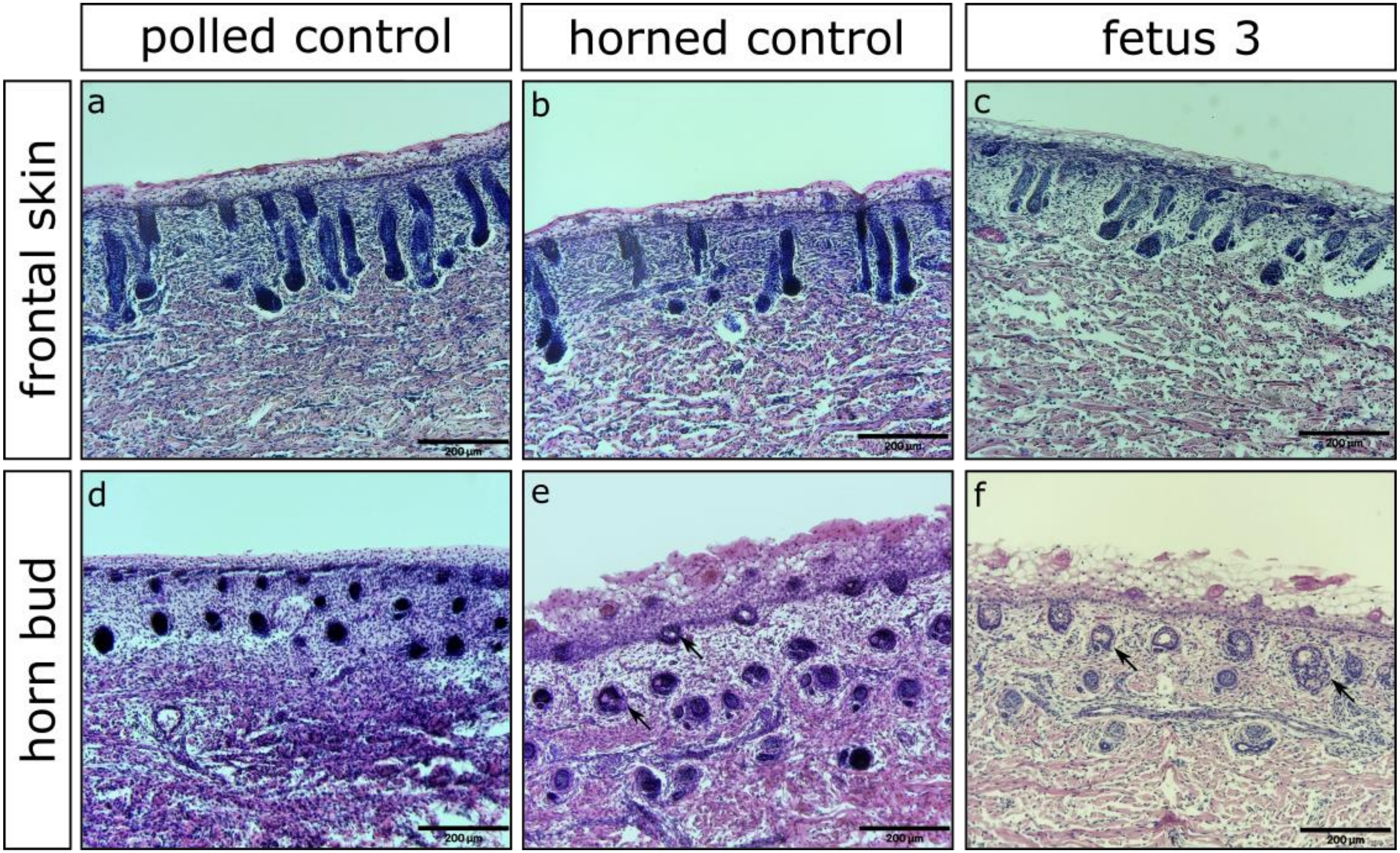

Supplementary Figure S6. Histological analysis of fetus 3 from embryo transfer 1 along with horned and polled control fetuses. (**a-c**) Frontal skin and (**d-f**) horn bud region of fetus 3 and age matched polled and horned controls at 151 days of gestation. Multiple layers of vacuolated keratinocytes can be seen in the horn bud region of horned fetuses along with sebaceous glands (black arrows). Stained with Hematoxylin and eosin.

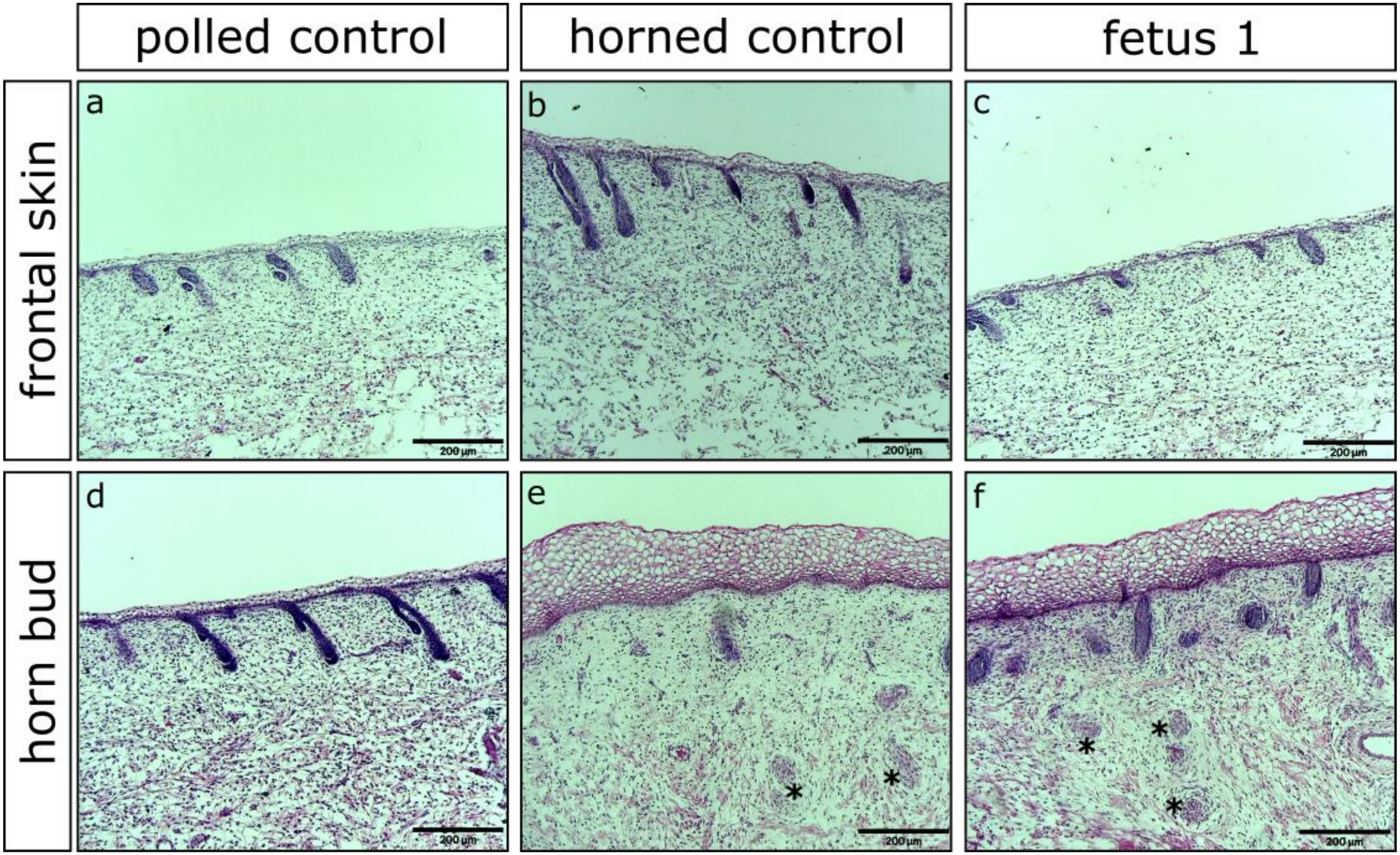

Supplementary Figure S7. Histological analysis of fetus 1 from embryo transfer 2 along with horned and polled control fetuses. (**a-c**) Frontal skin and (**d-f**) horn bud region of fetus 1 and age matched polled and horned controls at 123 days of gestation. Multiple layers of vacuolated keratinocytes can be seen in the horn bud region of horned fetuses along with thick nerve bundles (black stars). Stained with Hematoxylin and eosin.

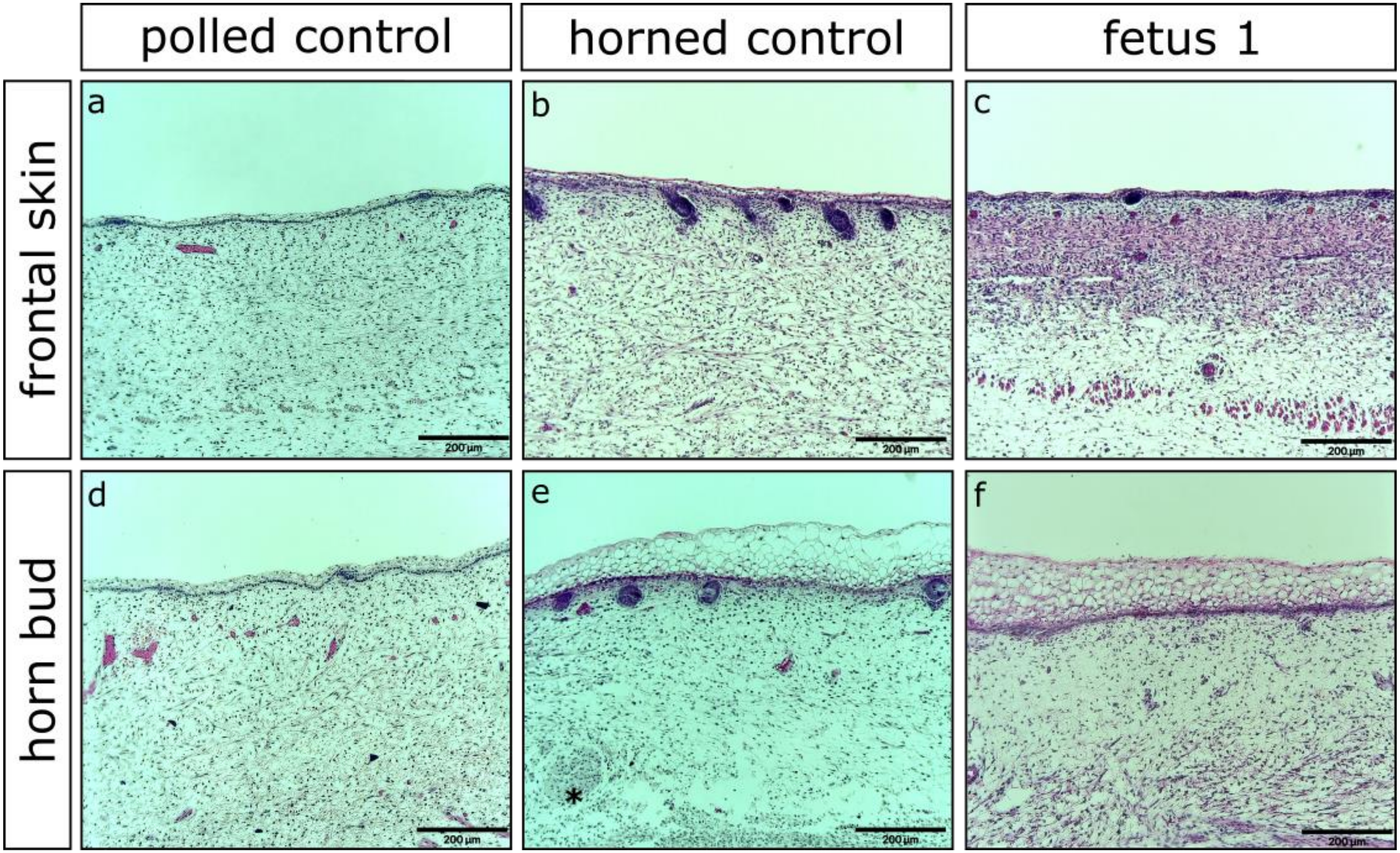

Supplementary Figure S8. Histological analysis of fetus 1 from embryo transfer 4 along with horned and polled control fetuses. (**a-c**) Frontal skin and (**d-f**) horn bud region of fetus 1 and age matched polled and horned controls at 95 days of gestation. Multiple layers of vacuolated keratinocytes can be seen in the horn bud region of horned fetuses along with thick nerve bundles (black stars). Stained with Hematoxylin and eosin.

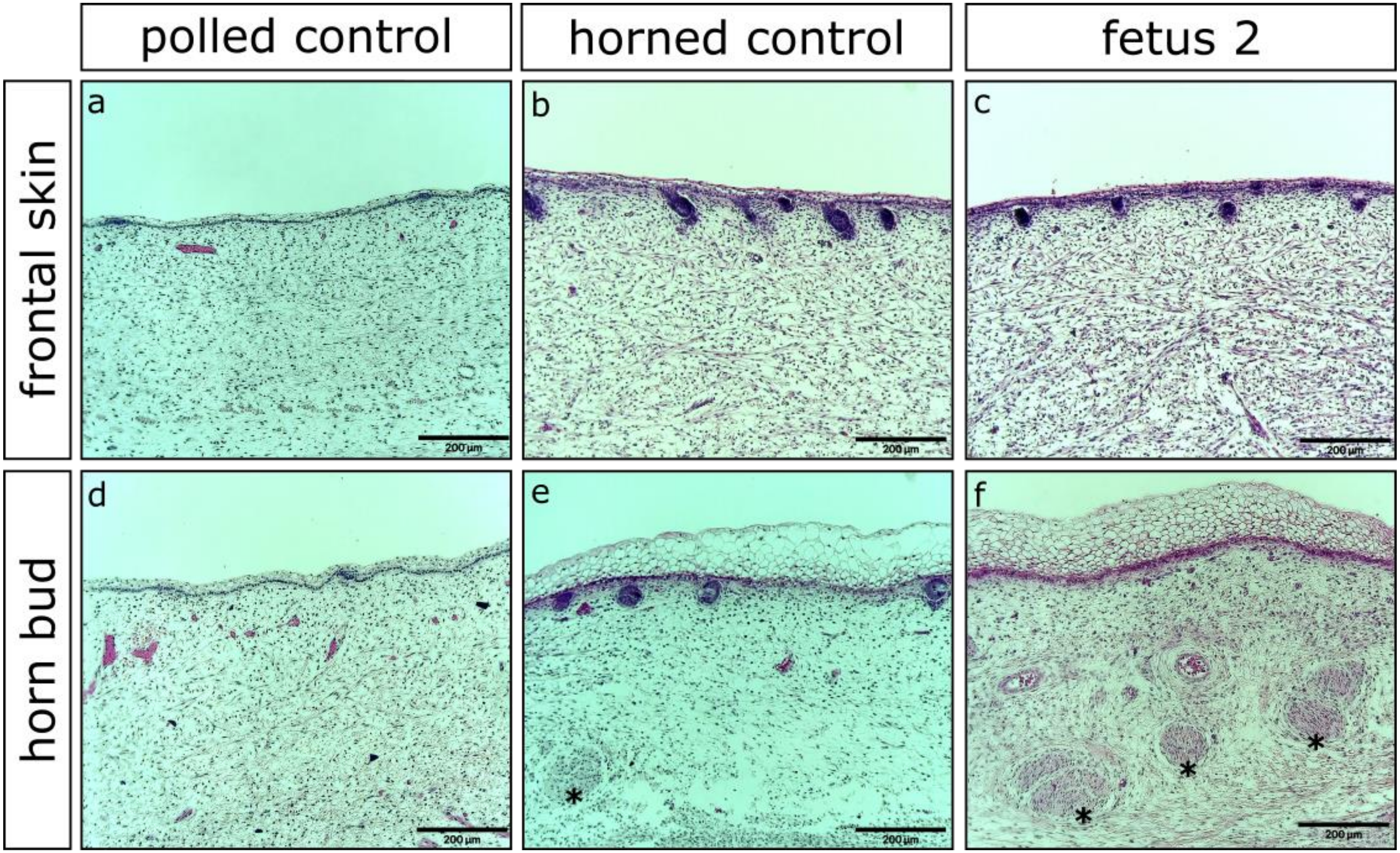

Supplementary Figure S9. Histological analysis of fetus 2 from embryo transfer 4 along with horned and polled control fetuses. (**a-c**) Frontal skin and (**d-f**) horn bud region of fetus 2 and age matched polled and horned controls at 95 days of gestation. Multiple layers of vacuolated keratinocytes can be seen in the horn bud region of horned fetuses along with thick nerve bundles (black stars). Stained with Hematoxylin and eosin.

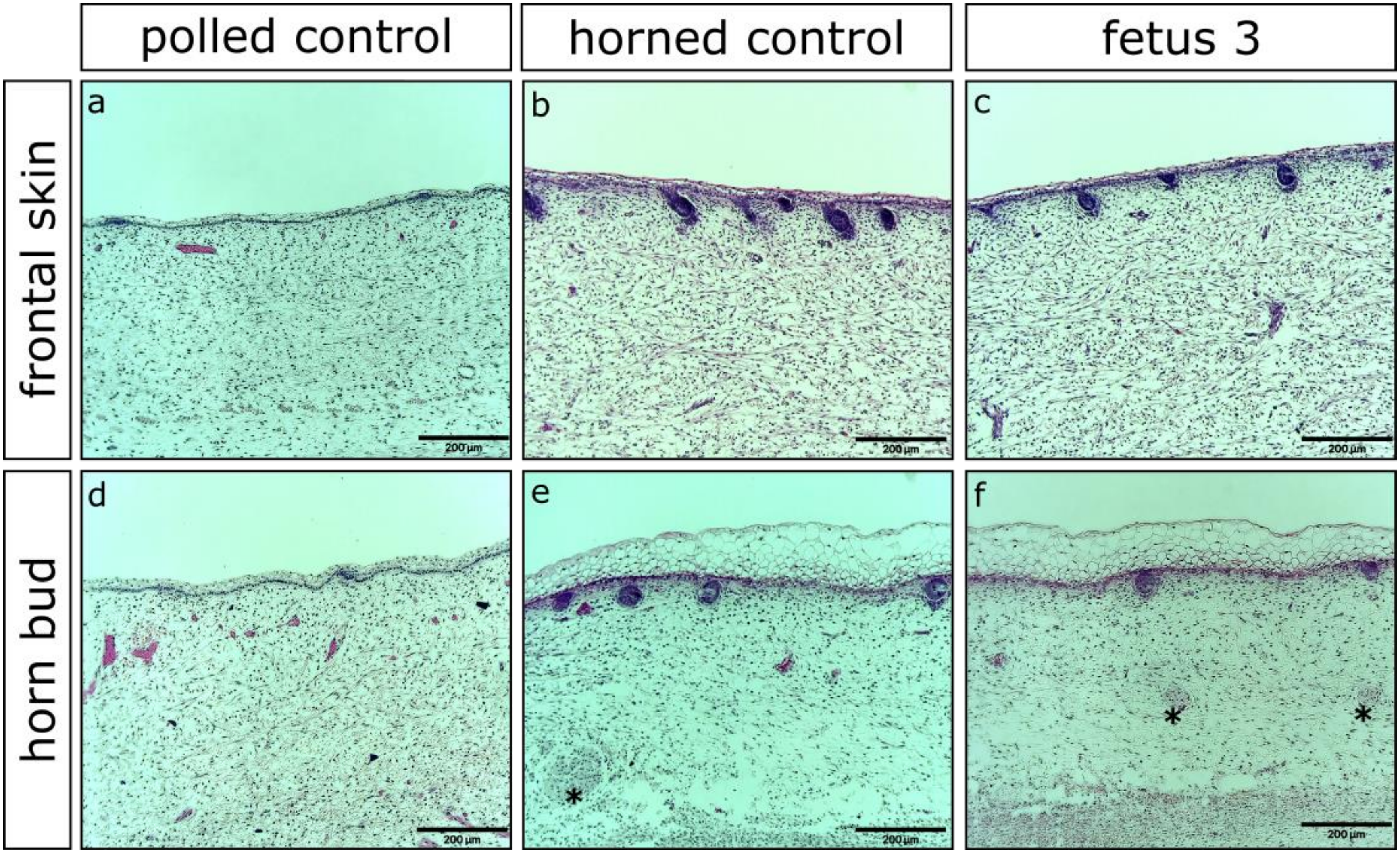

Supplementary Figure S10. Histological analysis of fetus 3 from embryo transfer 4 along with horned and polled control fetuses. (**a-c**) Frontal skin and (**d-f**) horn bud region of fetus 3 and age matched polled and horned controls at 95 days of gestation. Multiple layers of vacuolated keratinocytes can be seen in the horn bud region of horned fetuses along with thick nerve bundles (black stars). Stained with Hematoxylin and eosin.
